# Terahertz Metamaterial Sensor for Sensitive Detection of Citrate Salt Solutions

**DOI:** 10.3390/bios12060408

**Published:** 2022-06-13

**Authors:** Xinxin Deng, Yanchun Shen, Bingwei Liu, Ziyu Song, Xiaoyong He, Qinnan Zhang, Dongxiong Ling, Dongfeng Liu, Dongshan Wei

**Affiliations:** 1 School of Electrical Engineering and Intelligentization, Dongguan University of Technology, Dongguan 523808, China; 2111903179@mail2.gdut.edu.cn (X.D.); 2020022415@m.scnu.edu.cn (Z.S.); hxy@dgut.edu.cn (X.H.); zhangqn@dgut.edu.cn (Q.Z.); lingdx@dgut.edu.cn (D.L.); 2 School of Information Engineering, Guangdong University of Technology, Guangzhou 510006, China; liudf@gdut.edu.cn; 3 Information Engineering Institute, Guangzhou Railway Polytechnic, Guangzhou 510432, China; shenyanchun@gtxy.edu.cn; 4 School of Optical-Electrical and Computer Engineering, University of Shanghai for Science and Technology, Shanghai 200093, China; 211180045@st.usst.edu.cn

**Keywords:** citrate salt, terahertz spectroscopy, metamaterial sensor, frequency shift, refractive index

## Abstract

Citrate salts (CSs), as one type of organic salts, have been widely used in the food and pharmaceutical industries. Accurate and quantitative detection of CSs in food and medicine is very important for health and safety. In this study, an asymmetric double-opening ring metamaterial sensor is designed, fabricated, and used to detect citrate salts combined with THz spectroscopy. Factors that influence the sensitivity of the metamaterial sensor including the opening positions and the arrangement of the metal opening ring unit, the refraction index and the thickness of the analyte deposited on the metamaterial sensor were analyzed and discussed from electromagnetic simulations and THz spectroscopy measurements. Based on the high sensitivity of the metamaterial sensor to the refractive index of the analyte, six different citrate salt solutions with low concentrations were well identified. Therefore, THz spectroscopy combined with a metamaterials sensor can provide a new, rapid, and accurate detection of citrate salts.

## 1. Introduction

Citrate salts (CSs), comprising citric acid anions and metal cations, have been widely used in the food and pharmaceutical industries [1]. When CSs are used as food additives, they can be used as a formula for soft water drinks, antiseptic for milk products, and sweetness correctors for food to make food taste better and promote appetite. They can also be used as nutritional supplements [2,3]. An appropriate dosage of CSs is harmless to the human body and can enhance normal metabolism in the body [4]. When CSs are used as medicines, they have different functions. For instance, potassium citrate (K-citrate) can be used to prevent and treat kidney stones, to regulate uric acid and treat hypokalemia [5]; lithium citrate (Li-citrate) can be used as a mood stabilizer to treat irritability and depression [6]; ferric citrate (Fe-citrate) can reduce the level of FGF-23 and maintain the balance of calcium and phosphorus in patients with chronic kidney disease, and improve the cardiac function [7]; zinc citrate (Zn-citrate) can be used as an effective ingredient in toothpaste, which can effectively control dental plaque, and relieve gingival bleeding and inhibit the generation of dental stones [8]; calcium citrate (Ca-citrate) can be used as the calcium fortifying agent with the best absorption effect [9]; magnesium citrate (Mg-citrate) can be commonly used as a saline laxative for patients to prepare the gastrointestinal surgery, and it can also be used to treat constipation and rectal cancer [10]; however, excessive consumption of CSs may change intestinal flora and increase gastrointestinal burden, resulting in aggravating symptoms such as osteoporosis and nervous system disorders [11]. Therefore, an accurate dosage control of each CS in food and medicine is very important for human health and safety.

At present, the traditional identification methods of CSs include the neutralization method combined with a titration solution [12], the refractive index method using an Abbe refractometer [13], the absorbance ratio method using a thymol blue pH indicator [14], and the colorimetric method with FeCl_3_ [15]. In practice, however, each method has its own limitations. For example, the neutralization method using a titrant solution results in a large difference because the endpoint of the titration is determined only with a visual examination. The perchloric acid used in the refraction method needs to be recalibrated before use. The color of the PH indicator in the absorbance ratio method sometimes is not obvious. Although the operation of the FeCl_3_ colorimetric method is relatively simple, due to different complexation reaction speeds, the time to complete the reaction is greatly different, resulting in the depth of color will increase with the extension of time. Therefore, it is necessary to develop a novel method to detect citrate salts. In this study, we propose a new technique combining the THz spectroscopy and a metamaterial sensor based on asymmetric double-opening rings to accurately detect the low-concentration CSs in aqueous solutions.

Terahertz wave is a type of electromagnetic wave with a frequency of 0.1~10 THz between the infrared wave and the microwave [16]. In the past two decades, applications of terahertz technology have greatly grown in various fields, including, but not limited to, non-destructive testing and imaging [17], anti-terrorism and security inspection [18], and materials science [19], as well as communications [20]. Due to its prominent characteristics of low scattering, high penetrability and fingerprint spectrum, the terahertz spectroscopy technology has received great attention from the scientific community, in addition to undergoing rapid development in industrial applications.

Metamaterials [21,22,23], as a kind of periodic artificial composite materials, are usually composed of subwavelength metal structures made on dielectric or semiconductor substrates. Through precise designs, metamaterials can exhibit supernormal physical properties that natural materials do not have, such as negative refractive index, negative permeability, abnormal transmission, and super-absorption [24,25,26,27]. In addition, metamaterials have good local field enhancement electromagnetic properties, which are not derived from the physical properties of materials themselves but depend on artificially constructed structures [28]. Metamaterials can flexibly control the phase, amplitude, and polarization of THz waves, providing effective carriers for THz devices to realize their functions [29,30,31]. Since the resonant frequency response of metamaterials is closely related to the dielectric constant of the surrounding medium, by depositing the analyte on the surface of the metamaterial, the quantitative analysis of the analyte can be realized by monitoring the resonance frequency shift of the metamaterial [32]. The development of terahertz metamaterial sensors (TMSs) originated in 2004 by Yen et al. [33], who first demonstrated an SRR (split-ring resonator) structure in the THz band and observed an inductor–capacitor (LC) resonance. In 2014, Singh et al. [34] designed an array of asymmetric square split rings on a silicon substrate to excite a resonance with an ultrahigh quality factor and obtained a sensitivity of 36.7 GHz/RIU (RIU, refractive index unit). In 2017, Hu et al. [35] used a split-ring resonator metasensor of Al metal to realize real-time monitoring of the interaction between bovine serum albumin (BSA) and four drug molecules and extracted the refractive index and absorption coefficient before and after the chemical reaction. In 2020, Cheng et al. [36] designed an asymmetric Cu metal split-ring resonator to high-sensitively detect A/G and A/G + IgG protein samples. In 2021, Zhang et al. [37] designed a new electromagnetically induced transparency (EIT)-like THz metamaterial biosensor for highly sensitive detection and rapid analysis of glioma cells. Therefore, metamaterials are suitable as signal-enhancing carriers for the highly sensitive detection of biomolecules and biological samples. More applications of TMSs in analyte detection are well-provided in the latest review articles [38,39,40,41,42].

In this study, we combine the THz spectroscopy and an asymmetric metamaterial sensor to accurately detect and differentiate six different citrate salts. First, the metamaterial sensor is designed, and the factors that influence the sensitivity of the metamaterial sensor are analyzed via electromagnetic simulations. Then, the metamaterial sensor is fabricated according to the optimal parameters derived from simulations and THz spectroscopy measurements of the metamaterial sensor with or without citrate salt samples. Lastly, the THz spectroscopy measurement results are analyzed and discussed.

## 2. Simulation and Experimental Methods

### 2.1. Design of the Metamaterial Sensor

Since the asymmetric split-ring resonators can excite asymmetric subradiant resonances to present a sharp narrow spectrum with high-quality factors and are highly sensitive to the variation in the surrounding dielectric environment [34,36], in this study, a terahertz metamaterial sensor with an asymmetric double-opening ring array was designed. The sensor has a three-layer structure. The upper metal layer is gold with a conductivity of 4.09 × 10^7^ S/m and a thickness of 200 nm. The lower layer is quartz with a thickness of 500 μm. The intermediate layer uses an 8 nm thick chromium as a binder. To check the sensitivity of the metamaterial sensor, electromagnetics simulations in CST studio software were performed with normalized TE incidence (electric field parallel to the opening direction of the metal ring).

#### 2.1.1. Effect of the Opening Position of the Metal Ring

**Figure 1:**
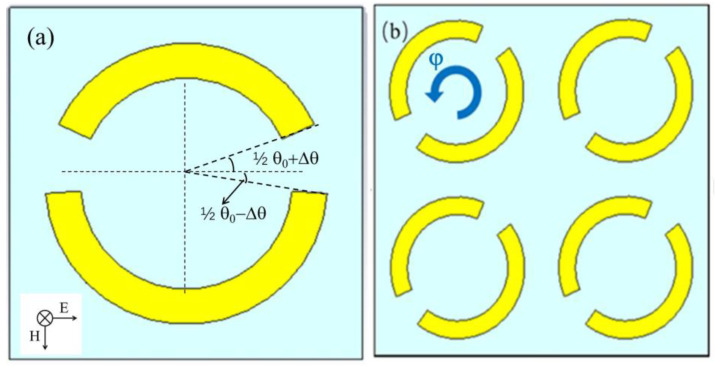
Schematic diagrams of the asymmetric metal ring unit with two openings at a changing angle of Δθ (**a**) and the asymmetric metal ring array at a rotation angle of φ = 40° (**b**). The THz wave TE incidence is indicated in the inset of (**a**).

To simulate the influence of the opening position of the metal ring on the sensitivity of the metamaterial sensor, metamaterials with different opening positions were designed and simulated. As shown in Figure 1a, the inner and outer radii of the ring were set to 18 μm and 24 μm, respectively, and the metal ring unit had two asymmetric sector openings, where the sector angle θ_0_ equaled 29°. By increasing the sector angle of the bottom half-ring and decreasing the sector angle of the top half-ring with the same changing angle of Δθ, a top–bottom asymmetric ring structure with two openings was obtained.

**Figure 2:**
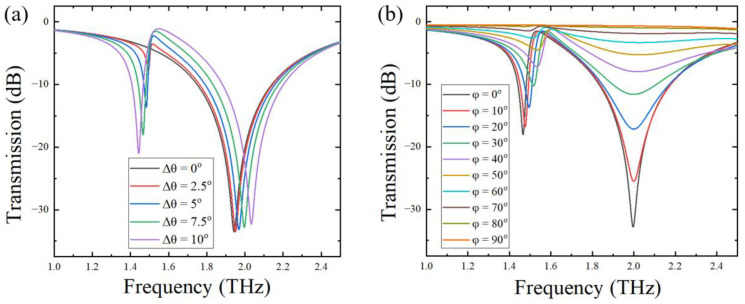
Transmission spectra of the metamaterial sensor with different changing angles (**a**) and with different rotation angles (**b**).

By changing the magnitude of Δθ, a series of asymmetric metal rings with different openings can be designed. Figure 2a shows the transmission spectra of the metal opening ring array metamaterial sensors with different changing angles of 0°, 2.5°, 5°, 7.5°, and 10°. It can be seen from the figure that, when the asymmetric opening structure appeared, i.e., Δθ > 0, two resonant peaks, one large and one small, appeared in the transmission spectrum of the sensor. For the sake of simplicity and accuracy, we choose the large resonant peak for analyses and discussion in what follows. The quality factor Q values according to the large resonant peak were 11, 12, 12, 13, and 13 for Δθ = 0°, 2.5°, 5°, 7.5°, and 10°, respectively, as shown in Table 1. The quality factor was defined as Q = f/FWHM, where f is the resonant frequency, and FWHM represents the full width at half-maximum of the resonant peak. The Q value reflects the resonance characteristics of the sensor. The larger the Q value, the sharper the transmission spectral curve, and the higher the sensitivity of the sensor. It can be seen from Table 1 that, when the changing angle Δθ gradually increased, the Q value slightly increased. When Δθ increased to 7.5°, the Q value reached the maximum. Therefore, Δθ = 7.5° was selected as the changing angle of the asymmetric opening ring structure for the fabrication of the metamaterial sensor.

#### 2.1.2. Effect of the Arrangement of the Metal Ring Units

To investigate the effect of the arrangement of the metal ring unit on the Q value of the sensor, we fixed the changing angle at Δθ = 7.5° and rotated the metal ring unit counterclockwise, with a rotating angle φ varying from 0° to 90°, with a step of 10°. In Figure 1b, the metal ring array structure with the rotation angles of 40° is shown. THz transmission spectra of the metal ring array sensor with different rotation angles were simulated and are shown in Figure 2b; the corresponding calculated Q values are displayed in Table 2. Since the resonant peak around 2.0 THz almost disappeared in the transmission spectra for φ > 60°, the resonant frequency and Q value data are not displayed in Table 2.

By comparing the above simulation data, it can be inferred that, as the rotation angle of the metal ring unit increased, the resonance peak slightly blue-shifted, the bandwidth of the resonant peak widened, and the calculated Q value decreased accordingly. Therefore, the rotation angle of φ = 0° showed an optimal sensitivity value.

### 2.2. Fabrication of Metamaterial Sensor

**Figure 3:**
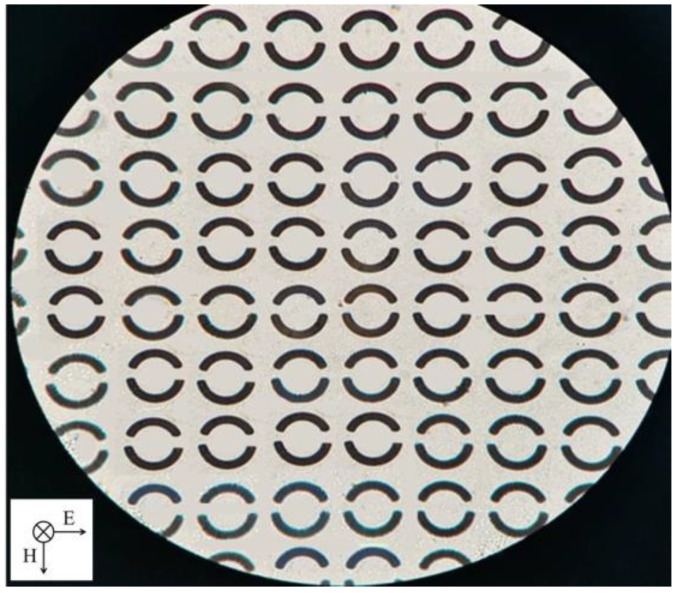
Optical microscope image of the sensor.

According to the above simulation results, in order to obtain a high Q value, the optimized changing angle of Δθ = 7.5° at openings and metal ring unit rotation angle of φ = 0° were chosen for sensor fabrication. The asymmetric double-opening ring array structure was fabricated on the 500 μm thick quartz crystal substrate using conventional photolithography. First, 8 nm Ti was deposited on the substrates, and then 200 nm Au was deposited on the Ti via the electron beam evaporation. Finally, the lift-off technology was implemented to remove the metallic layer on the photoresist. An optical microscope image of the fabricated sensor is shown in Figure 3. The period of the metal ring array is 60 μm × 60 μm, and the physical size of the sensor is 1.5 cm × 1.5 cm.

### 2.3. Terahertz Spectroscopy Measurements of Citrate Salt Samples

Citrate salt samples including the Li-, K-, Mg-, Ca-, Fe-, and Zn-citrates used in the experiment were all purchased from Maclin Company (Shanghai, China). All THz spectroscopy measurements in this research were performed using the transmission module of a THz–TDS spectrometer (Advantest Photonix, TAS7500SU, Advantest Corporation, Tokyo, Japan). Two types of THz spectroscopy measurements were performed: one is for the CS powder samples, and the other is for the CS solutions. For powder sample measurement, about 80 mg CS sample was weighed and ground into tiny powders with an agate mortar and then compressed into a pallet with a diameter of 13 mm and a thickness of around 0.55 mm, using a tablet press (Hefei Kejing Material Technology Co., Ltd., Hefei, China) under the working pressure of 10 MPa. To obtain accurate THz spectroscopy of these CS powder samples, each citrate salt powder sample was compressed into three pallets with a small thickness difference, and each pallet was analyzed with three repetitive measurements.

For the CS solution THz spectroscopy measurement, 6.13 mg CS powder was dried, weighed, and dissolved in 10 mL of deionized water in a tube with a mass concentration of 0.613 mg/mL. As the mass concentration prepared is less than the solubility of each CS, the CS solute can be completely dissolved in water. Before the CS solution measurement, the metamaterial sensor was soaked in anhydrous ethanol for half an hour and completely dried with the blow of nitrogen. Then, 120 μL CS solution was dropped on the surface of the sensor using a pipette and was evenly spread using a homogenizer. Afterward, the sensor with the deposited CS solution was put into an oven to dry at a temperature of 40 °C for about 5 h. Lastly, the THz spectroscopy measurement of the sensor covered with the dried CS sample was performed. All of the THz spectroscopy measurements were carried out at room temperature of 24.0 ± 1.0 °C and humidity of less than 40%.

## 3. Results and Discussion

### 3.1. Factors That Influence Sensitivity of the Metamaterial Sensor Covered with Analytes

**Figure 4:**
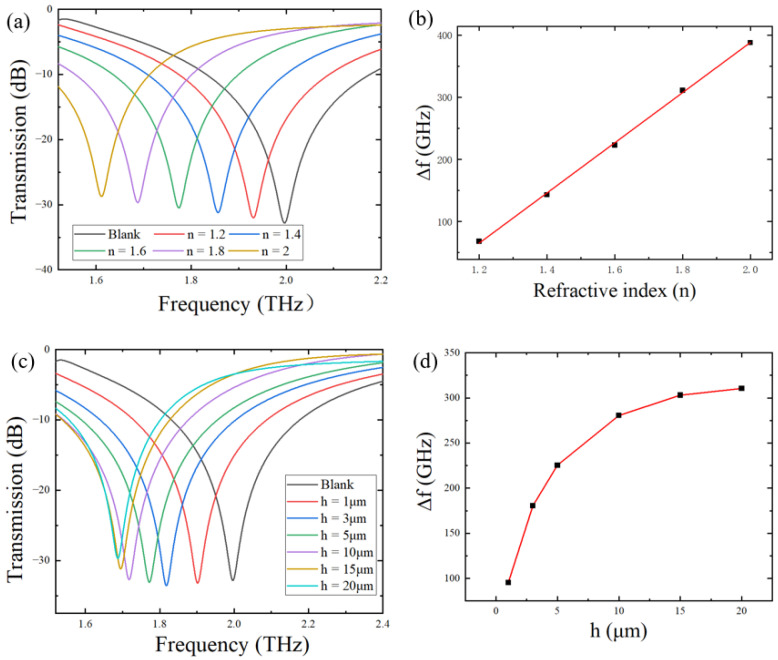
(**a**) Transmission spectra of the simulated metamaterial sensor covered by analytes with different refractive indices at *h* = 20 μm; (**b**) variation in the resonant peak frequency shift with the refractive index of the analyte; (**c**) transmission spectra of the simulated metamaterial sensor covered by analytes with different thicknesses at *n* = 1.8; (**d**) variation in the resonant peak frequency shift with the thickness of the analyte.

In order to explore the sensitivity of the metamaterial sensor for the detection of analytes, we first evaluated its sensing performance via simulations. In simulations, we chose to deposit an analyte with a thickness of 20 μm on the surface of the metamaterial sensor and analyzed the sensing performance of the sensor by changing the refractive index of the analyte deposited on it. Refractive indices of citrate salts are known to be around 1.6–2.0, so the refractive index of the simulated analyte is set in the range of 1.2–2.0. The simulated THz transmission spectra of analytes with different refractive indices are shown in Figure 4a. As the refractive index increased from 1.2 to 2.0 with a step of 0.1, the resonant frequency peak significantly red-shifted from 1.930 to 1.612 THz, compared with the blank metamaterial without any analyte, but the amplitude value did not change significantly. Since the sensing principle of the terahertz metamaterial sensor is to transform changes in dielectric parameters of the metamaterial environment into changes in electromagnetic spectral signals, it is necessary to examine the relationship between the resonance peak shift and the change in the electromagnetic parameter of the analyte. Figure 4b shows the variation in the resonant peak shift, ∆*f*, with changes in the refractive index *n*. A linear relationship between ∆*f* and *n* is easily detected. The sensitivity of the sensor S was defined as the slope of ∆*f* vs, and *n* was found to be 402 GHz/RIU, which is comparable to that of widely used metamaterials, as shown in Table 3.

In addition, the thickness of the analyte deposited on the surface of the metamaterial also has an important influence on the sensitivity of the sensor. Figure 4c shows that, when the thickness of the analyte, *h*, changed from 1 μm to 20 μm, the resonant peak frequency shift varied from 95.5 GHz to 310.5 GHz under the fixed refractive index of 1.8. In addition, as evident in Figure 4d, there was a nonlinear relationship between the thickness and the frequency shift. At h < 15 μm, the curve varied prominently relative to thickness, indicating that the sensor was sensitive to the thickness of the analyte. When the thickness gradually increased to 20 μm, the curve became flat, indicating that the increase in thickness left the frequency shift almost unchanged. In other words, Δf reached its limit value at h ≥ 20 μm.

### 3.2. THz Spectroscopy Detection of Citrate Salts Using the Metamaterial Sensor

**Figure 5:**
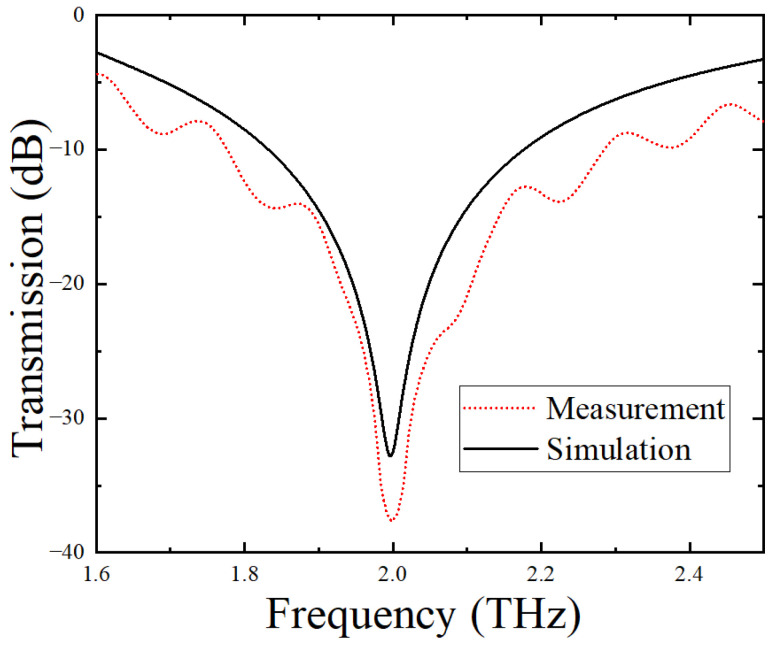
Measured and simulated THz transmission spectra of the blank sensor without analytes.

Before detecting different citrate salt samples, the THz spectrum of the blank metamaterial sensor without the CS analyte was measured and compared with the theoretical simulation result, as shown in Figure 5. From this figure, it can be seen that the featuring frequency peak of the blank sensor was 1.998 THz, which was completely consistent with the simulated resonance peak of 1.998 THz, indicating that the experiment and simulation results were in good agreement. In addition, the THz transmission intensity of the fabricated sensor at all frequencies other than those near the resonant peaks was estimated to be ~40% of the incident THz intensity, indicating that the sensor, like most THz asymmetric split-ring metamaterials, experienced a low loss.

**Figure 6:**
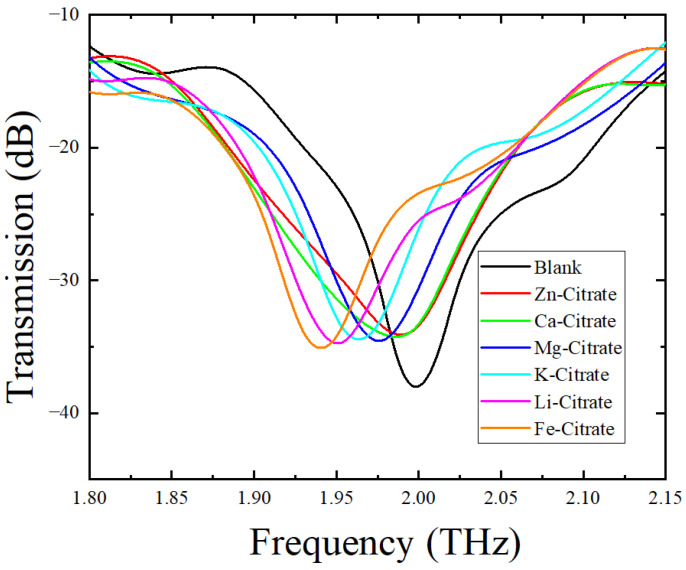
Transmission spectral curves of the blank sensor and the sensor covered with six different CSs.

In order to verify that the designed asymmetric double-slit-ring metamaterial sensor can distinguish different citrate salts, we measured the transmittance of the metamaterial sensor covered with different citrate salts. It can be seen from Figure 6 that there were six different featuring peaks in the transmission spectral curves of the six different CSs. Table 4 lists the featuring frequency and shift in resonant peak frequency (relative to the blank sensor) of the sensor covered with different citrate salts. Compared with the featuring peak of the blank sensor, all of featuring peaks of the sensor covered with CSs red-shifted, and the red-shift order of the CSs was Zn-citrate < Ca-citrate < Mg-citrate < K-citrate < Li-citrate < Fe-citrate. To explain the reason behind the resonant frequency red-shift of the sensor covered with CSs, an LC circuit [45,46] was used to define the resonance frequency of the metamaterial sensor with or without samples as

(1) $$f_{resonant}=\frac{1}{2\pi \sqrt{L_{eq}C_{eq}}}$$

where *L_eq_* is the equivalent inductance which mainly depends on the structure and size of the metamaterial sensor, and *C_eq_* is the equivalent capacitance which mainly depends on the dielectric parameters of the sample on the opening positions of the metal ring. Therefore, when the metamaterial sensor was covered with an analyte, *C_eq_* increased, and the resonant frequency decreased, which led to a larger redshift of the featuring peak.

**Figure 7:**
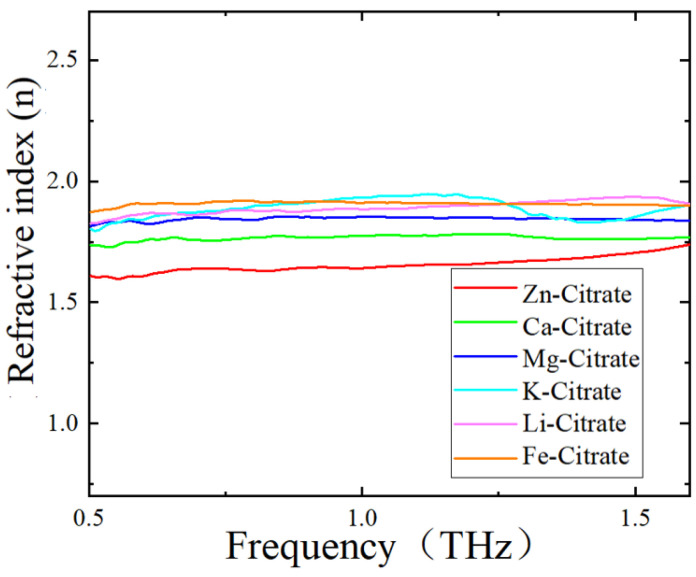
Refractive indices of CSs measured by THz–TDS system.

As for the different frequency shifts of the metamaterial sensor covered with different citrate salts, we ascribed the reason to the different refractive indices of these CSs. To verify this viewpoint, refractive indices of these six citrate salt samples were measured. Figure 7 shows the variations in the refractive index of CS samples with frequency. All of these six curves underwent a mild change with frequency in the range of 0.5–1.6 THz. The refractive index of each citrate salt sample was obtained by averaging the refractive index data around 1.0 THz and is shown in Table 5. We can see that the order of refractive indices was Zn-citrate < Ca-citrate < Mg-citrate < K-citrate < Li-citrate < Fe-citrate. Since the equivalent capacitance increased with the increase in the refractive index of the analyte deposited on the metamaterial, an increase in the refractive index would result in a decrease in resonant frequency, according to Equation (1). Therefore, when the analyte varied from Zn-citrate to Fe-citrate, the resonant frequency decreased and frequency shift increased, which was consistent with the results shown in Figure 6 and listed in Table 4. In addition, since the weights of the measured citrate salts are small and were also the same in this study, the difference in resonant frequency resulting from the thickness of the citrate salt deposited on the sensor was ignored. Therefore, combined with a metamaterial sensor, THz spectroscopy could well differentiate citrate salts according to their refractive index difference.

## 4. Conclusions

An asymmetric double-opening ring metamaterial sensor was designed, fabricated, and used to detect six citrate salt samples. The special asymmetric openings made the blank sensor have a main featuring frequency peak around 2.0 THz. Simulation results showed that the Q value of the asymmetric opening was higher than that of the symmetrical opening, and the maximal theoretical sensitivity reached 402 GHz/RIU. The Refractive index and thickness of the analyte deposited on the surface of the sensor had significant effects on the sensitivity of the sensor from theoretical simulations. With the increase in the refractive index of the analyte deposited on the sensor, the resonant frequency shift of the sensor covered with analytes relative to the blank sensor increased. When the thickness of the analyte was less than 20 μm, with the increase in thickness, the shift in resonant frequency increased. Based on the high sensitivity of the sensor to the analyte’s refractive index, six different citrate salt solutions were detected. Although the difference in refractive index between these six CSs was small, the THz spectroscopy, combined with the metamaterials sensor, was still able to well identify these citrate salts. Therefore, THz spectroscopy combined with a metamaterials sensor can provide a new, rapid, and accurate detection of citrate salts.

Despite the above-revealed success in the detection of citrate salt solution samples by combining THz spectroscopy with the metamaterial, the concentrations of citrate salt solution samples were ~1 mM, which may be much higher than a concentration appropriate for practical use. Trace detection of biological samples under physiological conditions and clinical uses continues to pose challenges in terms of sensitivity in THz sensors. Metamaterials coupled with graphene [47], microfluidic channels [48], and photonic crystals [49,50,51,52,53,54] may be very promising routes for super-highly sensitive THz spectroscopy detection of biological samples in the future.

## Figures and Tables

**Table 1:** Resonant frequency and Q value of the metamaterial sensor with different changing angles.

Δθ	Resonant Frequency (THz)	Q Value
0°	1.943	11
2.5°	1.950	12
5°	1.968	12
7.5°	1.998	13
10°	2.035	13

**Table 2:** Resonant frequency and Q value of the metamaterial sensor with different rotation angles.

Rotation Angle	Resonant Frequency (THz)	Q Value
0°	1.998	13
10°	2.000	8
20°	2.000	5
30°	2.000	3
40°	2.019	3
50°	2.028	2
60°	2.028	2

**Table 3:** Sensitivity comparison between the proposed metal metamaterial with widely used ones.

Structure	Sensitivity (GHz/RIU)	Detection Target	Metal and Reference
Asymmetric split-circle ring	402	Citrate salts	Au, this work
Asymmetric split-square ring	36.7	Photoresist	Al [34]
Asymmetric split-circle ring	240	Protein	Cu [36]
Asymmetric split-square ring	387	Carcinoembryonic antigen	Au [43]
Concentric square rings and a cylinder positioned at their center	360	Thin films	Ag [44]

**Table 4:** Featuring peak positions and frequency shifts of the metamaterial sensor covered with different CSs.

Name	Resonant Frequency (THz)	Frequency Shift (GHz)
Zn-citrate	1.989 ± 0.002	9
Ca-citrate	1.985 ± 0.004	13
Mg-citrate	1.976 ± 0.003	22
K-citrate	1.961 ± 0.005	37
Li-citrate	1.950 ± 0.005	48
Fe-citrate	1.945 ± 0.006	56

**Table 5:** Refractive index data of CSs.

Zn-Citrate	Ca-Citrate	Mg-Citrate	K-Citrate	Li-Citrate	Fe-Citrate
1.65 ± 0.03	1.76 ± 0.01	1.84 ± 0.07	1.88 ± 0.03	1.89 ± 0.01	1.91 ± 0.06

## Data Availability

Not applicable.
